# The effectiveness of a multidisciplinary QI activity for accidental fall prevention: Staff compliance is critical

**DOI:** 10.1186/1472-6963-12-197

**Published:** 2012-07-12

**Authors:** Sachiko Ohde, Mineko Terai, Aya Oizumi, Osamu Takahashi, Gautam A Deshpande, Miwako Takekata, Ryoichi Ishikawa, Tsuguya Fukui

**Affiliations:** 1 Center for Clinical Epidemiology, St. Luke’s Life Science Institute, Tokyo 104-8560, Japan; 2 Office of Medical Safety Management, St. Luke’s International Hospital, Tokyo, Japan; 3 Medical Information Center, St. Luke’s International Hospital, Tokyo, Japan; 4 Department of General Internal Medicine, St. Luke’s International Hospital, Tokyo, Japan; 5 Department of Internal Medicine, University of Hawaii, Honolulu, Hawaii; 6 Department of Neurosurgery, St. Luke’s International Hospital, Tokyo, Japan; 7 St Luke’s International Hospital, Tokyo 104-8560, Japan

**Keywords:** Accidental falls, Fall prevention, QI activities, High compliance rate, Inpatients

## Abstract

**Background:**

Accidental falls among inpatients are a substantial cause of hospital injury. A number of successful experimental studies on fall prevention have shown the importance and efficacy of multifactorial intervention, though success rates vary. However, the importance of staff compliance with these effective, but often time-consuming, multifactorial interventions has not been fully investigated in a routine clinical setting. The purpose of this observational study was to describe the effectiveness of a multidisciplinary quality improvement (QI) activity for accidental fall prevention, with particular focus on staff compliance in a non-experimental clinical setting.

**Methods:**

This observational study was conducted from July 2004 through December 2010 at St. Luke’s International Hospital in Tokyo, Japan. The QI activity for in-patient falls prevention consisted of: 1) the fall risk assessment tool, 2) an intervention protocol to prevent in-patient falls, 3) specific environmental safety interventions, 4) staff education, and 5) multidisciplinary healthcare staff compliance monitoring and feedback mechanisms.

**Results:**

The overall fall rate was 2.13 falls per 1000 patient days (350/164331) in 2004 versus 1.53 falls per 1000 patient days (263/172325) in 2010, representing a significant decrease (p = 0.039). In the first 6 months, compliance with use of the falling risk assessment tool at admission was 91.5% in 2007 (3998/4368), increasing to 97.6% in 2010 (10564/10828). The staff compliance rate of implementing an appropriate intervention plan was 85.9% in 2007, increasing to 95.3% in 2010.

**Conclusion:**

In our study we observed a substantial decrease in patient fall rates and an increase of staff compliance with a newly implemented falls prevention program. A systematized QI approach that closely involves, encourages, and educates healthcare staff at multiple levels is effective.

## Background

Accidental falls among inpatients are a substantial cause of hospital injury, resulting in extended lengths of stay and a decline in quality of life [1-3], and occur in 3-20% of inpatients [4]. Starting on October 1, 2008, the United States’ Center for Medicare and Medicaid Services began including inpatient falls as a‘never event’—i.e., a preventable condition that should not occur after admission to the hospital [4]. Given that hospital falls are a universal occurrence, many of which are preventable, in-hospital falling remains a major healthcare concern internationally [5]. In recent randomized controlled trials, risk assessment tools and intervention plans have often been combined and studied as part of multifactorial intervention programs which include various modalities, including exercise, medication modification, vitamin D supplementation, environment/assistive technology, and both staff and patient education [6-10]. Though data from randomized controlled trials and high-quality systematic reviews are considered the highest level of evidence, the evidence-practice gap remains substantial [11-13]. Although a number of successful experimental studies on fall prevention have been published, the degree of effectiveness varies across studies [7-9]. This is even more surprising given the many similarities among multidisciplinary fall prevention protocols. In an non-experimental clinical setting, Schwendimann et al., conducting an observational study to investigate the effect of an interdisciplinary falls prevention program, failed to show a substantial decrease in either frequency of falls or consequent injuries, citing low staff compliance as a probable cause [14]. While this suggests the importance of staff compliance with effective, but potentially cumbersome, multifactorial interventions, the role of staff compliance with prevention protocols has not been fully investigated in a routine clinical setting.

Quality improvement (QI) activity typically includes staff education, serial audit and feedback, and may even include financial incentives. It includes multiple modalities, but is often most successful when it employs a multidisciplinary, continuous, and systematic approach to hospital quality and care [15]. In particular, high rate of compliance with intervention plans may be achieved through serial audit and feedback sessions [16]. At our hospital prior to 2004, assessments and intervention plans varied from ward to ward and were subject to neither strict monitoring nor standardized reporting. The purpose of this observational study was to assess the efficacy of a multidisciplinary QI activity for accidental fall prevention, with particular focus on staff compliance in a routine, non-experimental, clinical setting.

## Methods

### Design, setting and sample

This observational study was conducted from July 2004 through December 2010 at St. Luke’s International Hospital, a 520-bed community-based, tertiary-level, teaching hospital in Tokyo, Japan, seeing an average of 461 inpatients per day with an average length of stay of 10.2 days. All adult inpatients, with the exception of maternity, preventative health screening patients, and intensive care patients, were included in the study. Ethical approval was obtained from the Research Ethics Committee of St. Luke’s International Hospital, Tokyo, Japan (approval code: 12-R003).

### Staff involved in QI activities

Three in-hospital groups, the fall prevention Working Group, Quality Indicator Committee (QIC), and Patient Safety Committee (PSC), were closely involved in QI activities. In 2006, taking into consideration the frequency of inpatients falls, our institution began a hospital-wide initiative to eliminate them. A multidisciplinary, voluntary working group was organized and consisted of a nurse safety manager, health information manager, clinical researcher, and other staff including doctors, nurses, physical and occupational therapists, and administration staff interested in falling prevention. While each member of the group was given a role, no extra compensation was given for participation and no dedicated staffs were hired explicitly to serve on the Working Group. The Working Group’s responsibility included evidence-based literature reviews of past protocols; development and analysis of a risk assessment tool, development of risk-specific intervention protocols and prevention strategies, and assessment of ward environmental safety issues.

QIC and PSC, also voluntary, multidisciplinary committees, were primarily responsible for monitoring inpatient fall events and providing feedback to departments, ward sections, and individual staff members. PSC was responsible for reviewing all incident cases occurring in the prior 24 hours. Based on this short-term monitoring, PSC then coordinated immediate feedback to appropriate departments, wards, and individuals. For long-term monitoring, QIC was responsible for collating and reviewing the monthly inpatient fall rate, in addition to providing departmental and ward feedback and public reporting. Several staff in the Working Group also participated in the PSC and QIC as well, thus facilitating communication and coordination of activities across groups.

Details of all groups, as well as responsibilities, are summarized in Table 1.

**Table 1:** Staffs of the QI activity for falling prevention

**Groups and Committees**	**Roles**	**Frequency**	**Staff**
Working Group	· Evidence-based literature reviews	Monthly	Safety manager (1), clinical researcher (1), health information manager (1), ward nursing managers (7), physicians (8), pharmacist (1), physical/occupational therapist (2).
	· Develop and validate risk assessment tool		
	· Develop intervention plans		
	· Assess specific environmental safety interventions		
QI Committee	· Review all quality indicators.	Monthly	Safety manager (1), clinical researcher (1), health information manager (6), ward nursing managers (6), physicians (20), pharmacist (1), administration staff (1)
	· Provide long-term monitoring and assist with department and ward feedback.		
	Publicly report QI data.		
Patient Safety Committee	· Review all incidents reported over prior 24 hours.	Everyday	Ward nurse managers (4), safety manager (1), attending physicians (3.ER, surgery, and internal medicine), pharmacist (2).
	· Provide immediate feedback to appropriate staff.		

### Fall prevention QI activities

QI activities for falling prevention at our institution include 1) the fall risk assessment tool, 2) an intervention protocol to prevent in-patient falls, 3) specific environmental safety interventions, 4) scheduled staff education, and 5) healthcare staff compliance monitoring and feedback mechanisms. A summary of all QI activities is shown in Table 2.

**Table 2:** Components of the QI activity for falling prevention

	**Status**	**Responsible Staff**
1. The fall risk assessment tool	Tool development	Working Group*^1^
	Application to clinical practice	Admission nurses
	Charting assessment results into electronic medical record (EMR)	Admission nurses
2. The intervention protocol	Intervention protocol development	Working Group
	Application to clinical practice	Ward nursing staff
	Charting intervention into EMR	Ward nursing staff
3. Specific environmental safety interventions	Ward-specific reviews and environmental intervention planning	Working Group
	Applying plans to wards	Staff of Facilities Dept.
4. Staff education	Developing education program	Working Group
	Participants	Ward nurses (yearly), new employees (orientation)
5. Healthcare staff compliance monitoring and feedback mechanisms	Data collection	Health information managers
	Report review (monthly)	QI committee*^2^
	Report review (daily)	Patient Safety Committee*^3^
	Provide feedback to ward nurse managers	Safety managers
	Provide feedback to ward nurses	Ward nurse managers

#### The fall risk assessment tool

First, the Working Group reviewed the literature to identify risk factors for falling, selecting 13 potential risk factors including history of falls, gait deficit, dizziness, inability to call for nursing assistance due to self-overestimate of ability, wandering, subjective nurse assessment of falling likelihood, use of sedating medications, gait assist device use, egestion disorders, intubation, vision disturbance, mental disorder, and disorientation. Admission nurses prospectively applied the 13-item fall risk assessment tool to 5700 inpatients within 24 hours after admission between November 1st, 2007 and April 30th, 2008. Admission nurses recorded assessment results into the electronic chart, with nurse managers on each ward auditing all entries.

Based on a multivariate logistic regression analysis, history of falls, gait deficit, dizziness, inability to call for nursing assistance due to self-overestimate of ability, wandering, subjective nurse assessment of falling likelihood, and use of sedating medications were found to be significant risk factors and included in the subsequent version of the risk assessment tool (AUC = 0.81; 95% CI: 0.74-0.87).

Data was re-analyzed yearly to reevaluate validity. Per analysis of 5668 patients admitted from May 1st, 2008 to November 30th, 2008 and 5777 patients admitted from May 1st, 2009 to November 30th, 2009, the AUC of this 7-item prediction model was 0.84 (95%CI: 0.79-0.89) and 0.79 (95%CI: 0.73-0.84), respectively. Thus, we confirmed that this risk assessment tool discriminated fallers from non-fallers in our hospital with acceptable accuracy. A summary of the falling risk assessment tool is shown in Table 3.

**Table 3:** Components of risk assessment tool and corresponding intervention plan

**Falling risk assessment tool**	**Intervention plan**
1. History of falls (Yes/No)	Base plan and I-A
2. Gait deficit (Yes/No)	I-A
3. Dizziness (Yes/No)	I-A
4. Inability to call for nursing assistance due to self-overestimate of ability (Yes/No)	I-B or II
5. Subjective nurse assessment of falling likelihood (Yes/No)	Base plan and I-A
6. Use of sedating medications (Yes/No)	III
·Patients with multiple risk factors provided intervention plan II.	

#### The intervention protocol to prevent in-patient falls

After developing the fall risk assessment tool, the Working Group reviewed previous studies and created an intervention plan based on relevant risk factors. Those having at least one risk factor were considered ‘high risk fall patients’ and written educational material raising awareness about the risk for falling was provided to these patients and their families, along with mounting educational posters at the bedside in appropriate rooms. Both educational material and bedside posters were individually tailored to each patient based on applied risk factors, following a previous study [7]. Additionally, nurses were encouraged to attach motion-alert devices on all patients identified as being unable to call for nursing assistance due to self-overestimate of ability on the risk assessment tool. Corresponding risk assessment and intervention plans are show in Table 3. All intervention plans for high risk patients were recorded on the electronic chart by nursing staff.

#### Specific environmental safety intervention

Per previous studies, as well as clinically-relevant input from nurses in the Working Group, many falls occurred in the toilet area [17,18]. Safety nurse managers and ward nurse managers reviewed all bathrooms in the hospital, and additional hand rails were subsequently installed in these areas, as well as around bed areas. Stand-alone, portable hand rails requiring no special installation (“best position bars”), were utilized, allowing added railing access at a relatively low cost. In addition, height discrepancy in flooring, such as step-offs between bathroom and bedroom, were removed. All construction was done between August 2007 and October 2007.

#### Staff education

A 60-minute education program was provided yearly to all clinical staff involved in patient care by members of the Working Group. The educational program included tutorials on how to apply an appropriate intervention plan based on results of the risk assessment tool, as well as a review of the most recent evidence from international studies of accidental inpatient falls, and a review of recent hospital fall events. Besides this regular education program, all newly hired staff were provided a 60-minute lecture about fall prevention as part of their orientation programming.

#### Healthcare staff compliance monitoring and feedback mechanisms

Short term monitoring of staff compliance was performed by the PSC, which met daily to review all incidents reported in the prior 24 hours. After identification and discussion of preventable events, feedback was provided to the nurse managers of each ward as well as nursing staff individually. Committee members discussed if cases were preventable and if events occurred due to inappropriately applied or absent intervention protocols. Members of PSC collected information if needed, directly from the staff that were in charge of fallen patients, for example, whether ward staffing was sufficient, or whether any remaining risk factor for recurrence existed.

Long term monitoring of staff compliance was performed by the QIC, which was responsible for reviewing 1) assessment rates at admission using the risk assessment tool, 2) rates of implementation of a risk-appropriate intervention plan, and 3) consequent injury rates. Data was compiled on a monthly basis for QIC review and discussion. QIC responsibilities also included assisting the PSC with providing feedback if staff compliance with fall risk assessment and intervention protocols was found to be low, and to discuss measures for improvement in these cases. In addition, QIC publicly reports all QI findings annually, both in print and electronically published formats.

### Data collection, measurement and statistics

Data were collected before, during, and after implementation of our multidisciplinary QI activities for falling prevention (2004–2010) on all adult inpatients, with the exception of maternity, preventative health screening patients, intensive care patients, and patients who stayed in hospital less than 24 hours. Patient characteristics such as gender, age, length of stay (LOS), and diagnosis were collected from the electronic medical record.

A fall was defined as “an event whereby an individual comes to rest on the ground or another lower level, with or without loss of consciousness.” [19] All inpatient falls were reported to the safety nurse manager within 24 hours by the first-on-scene healthcare staff, using a standardized fall incident reporting form. To mitigate the risk of under-reporting, any staff encountering a fall incident is required to write a report, even if he/she was not the primary person responsible for the fallen individual’s care or documentation. Reports included event location, circumstances surrounding the fall, patient demographics, presence and extent of injuries, as well as admission risk assessment and corresponding interventions implemented.

Overall fall rates per 1,000 patient days were calculated using falls as the numerator and patient days as the denominator. Staff compliance data was collected by health information managers via the electronic medical record. Compliance with risk assessment was calculated using the number of patients with recorded risk assessments as the numerator and the total number of adult patients all adult inpatients (with the exception of maternity, preventative health screening patients, intensive care patients, and patients who stayed in hospital less than 24 hours) as the denominator. Staff compliance with intervention plan implementation was calculated using the number of patients with appropriate intervention plans recorded in the electronic medical record as the numerator and the total number of high risk patients having at least one risk factor as the denominator.

In order to assess the year-over-year progress of the QI activity, Chi-square trend test was used to model the rate of falls per 1,000 patient days from 2004 to 2010. STATA software version 10.0 (College Station, Texas, USA) was used for all statistical analyses.

## Results

During our 7-year study period, a total of 109816 patients were hospitalized. Among these, 71396 patients remained for risk assessment after exclusion of maternity, preventative health screening patients, intensive care patients, and patients who stayed in hospital less than 24 hours. Since the initiation of standardized risk assessment in 2007, 16829 patients (23.6%) were identified to be at risk for falls. Patient characteristics, event, and compliance data are summarized in Tables 4 and 5.

**Table 4:** Intervention plan

**Plan**	**Intervention**
**Base plan**	Consider bed height, bed rail and other environmental safety measures.
**I-A**	Patients asked to utilize handrails.
	Patients asked to call nurse for assistance with mobility.
**I-B**	Patients attached to motion sensor.
	Nurse assists patients while toileting
**II**	Nursing staff and physicians meet regarding patient-specific fall plans.
	Patients attached to motion sensor.
	Nurse assists patients while toileting.
	Use of additional bed rails.
	Family asked to assist if possible; if not possible, patients moved to nursing station for direct observation.
**III**	Motion sensor (night only)
	Patients asked to utilize handrails.

**Table 5:** Prevalence of falls and fall-related injuries from 2004 to 2010

	**2004**	**2005**	**2006**	**2007**	**2008**	**2009**	**2010**
Number of patients admitted to the hospital	164,331	165,662	170,402	171,791	167,492	172,992	172,325
Numbers of males (%)	80,077 (48.7)	79,254 (47.8)	81,227 (47.7)	83,028 (48.3)	82,573 (49.3)	83,405 (48.2)	84,297 (48.9)
Mean of age (SD)	53.9(26.5)	54.4(26.4)	54.1(26.4)	54.6(27.0)	55.6(26.3)	55.3(26.7)	55.8(27.0)
Mean of length of staying(SD)	12.5(42.9)	11.5(25.3)	11.0(41.7)	11.1(66.0)	10.5(29.2)	10.1(19.0)	10.2(19.5)
No. of patients after exclusion criteria	8,640	9,392	10,108	10,554	10,714	11,209	10,779
Assessment rate of new patients (%)	No assessment done			4068/4471 (91.0*)	104149 (94.7)	11860 (96.9)	10512 (97.5)
Patients who had at least one risk factor	No assessment done			1942	4463	5014	5410
Number of falls	350	336	353	302	252	252	263
Number of repeated fallers	39	33	60	40	30	17	39
Numbers of fracture from fall	6	3	8	5	7	11	4
Fall rate (‰) (95‰CI)	2.13 (1.91-2.36)	2.03 (1.82-2.26)	2.07 (1.86-2.30)	1.76 (1.57-1.97)	1.50 (1.32-1.70)	1.46 (1.28-1.65)	1.53 (1.34-1.72)
Fracture rate from fall	0.04	0.02	0.05	0.03	0.04	0.06	0.02
(‰)(95‰CI)	(0.01-0.08)	(0.01-0.05)	(0.02-0.09)	(0.01-0.06)	(0.01-0.08)	(0.03-0.11)	(0.01-0.05)

* Data is room August to December because assessment rate of new patients were collected from August 2007.

The overall fall rate was 2.13 falls per 1000 patient days (350/164331) in 2004 versus 1.53 falls per 1000 patient days (263/172325) in 2010, representing a significant decrease (p = 0.039). Bone fracture rates due to falls among hospitalized patients declined, though not significantly, from 0.04 fractures per 1000 patient days (6/164331) in 2004 to 0.02 fractures per 1000 patient days (4/172325) in 2010. Compliance with use of the falling risk assessment tool at admission was 91.5% in 2007, representing the latter half of the year with assessments beginning in July, and increased to 97.6% in 2010. The rate of implementation of an appropriate intervention plan was 89.6% in 2007, increasing to 95.3% in 2010. While this varied substantially by ward in 2007, all wards achieved close to 100% compliance (range: 98.5%-100%) in 2010.

## Discussion

This observational study assessed fall rate over time, consequent injuries, and characteristics of hospitalized patients in the periods before and after QI activity implementation. In addition, staff compliance with risk assessment on admission, as well as implementation of risk-stratified intervention plans, was examined. Before QI activities were implemented at our facility in 2007, the accidental fall rate had remained constant at approximately 2.00 falls per 1000 patient days. This study documents a 25% reduction of inpatient falls over five years, from 2.13 falls per 1000 patient days in 2004 to 1.53 falls per 1000 patient days in 2010, with the most dramatic reduction from 2006 to 2009. The consequent injury rate was less than 0.1% for any year. Feasibility of this integrated approach was excellent, as reflected by high staff compliance with use of assessment tools and implementation of intervention strategies across wards and departments.

Our initial fall rate at the beginning of the study period was lower than other studies in urban, acute-care hospitals, though in line with the lower end of the reported spectrum, typically between 2.2 falls per 1000 patient days to 6.3 falls per 1000 patient days [20-22]. While the interventions put in place in 2006 were effective, aiding the further decrease was that the assessment rate of newly admitted patients was nearly 100% in 2010. Even if a robust preventive program exists, effectiveness is unlikely if compliance is low. A previous, observational study by Schwendimann et al. failed to show a substantial decrease in either frequency of falls or consequent injuries following the implementation of a similar interdisciplinary fall prevention program; the authors state that this was likely due to low staff compliance [14]. In contrast, a 2010 randomized controlled trial by Dykes et al. achieved a 46% reduction in their intervention group compared to a 25% reduction in the control group. Their staff compliance rate with intervention was 81%, corroborating the success in our study, which saw a final compliance rate of 95.3% [7]. This suggests that robust interventions themselves are necessary--but not sufficient--for fall prevention. Staff compliance with effective interventions is a critical, and perhaps often overlooked, factor in further closing the evidence-practice gap.

To this end, our QI activity included three elements to purposefully promote staff compliance with the fall prevention protocol. First, the Working Group in our hospital systematically involved healthcare staff on both planning and implementation levels in a multidisciplinary strategy to address the problem of accidental falls. The involvement of staff from multiple clinical and ancillary disciplines at the highest levels of planning may have influenced compliance at later points “downstream.” Second, all fall events were systematically documented, and subsequently followed by consistent short- and long-term auditing and feedback provided to hospital, department, ward, and individual staff at regular intervals. Finally, evidence-based reviews and practical training in protocol use were provided regularly to all staff in the form of repeated, evidence-based educational programming.

This study has some limitations. First, there was too little data on bone fractures after falls, thus we could not investigate the influence of our multidisciplinary QI activity on the rate of reduction of bone fractures. Second, this study was conducted in a single institution in a Japanese acute care hospital, with consequent uncertainty about generalizability. Future multi-center studies with larger numbers of patients may allow us to better clarify both effectiveness in reducing consequent injury, as well as assess the feasibility of our QI activity and result generalizability to other hospitals. The goal of our study was to decrease inpatients fall hospital-wide. Application of this protocol to those at highest risk, such as inpatients on geriatric or long-term nursing care wards, may be useful. Finally, it is difficult to distinguish the role that compliance plays in effective QI activity versus elements of the activity itself. Our initial compliance rate was already high and this may not be the case at other institutions. Our results, however, suggest that both are necessary components of QI protocols and should be maximized during planning phases. While we achieved a substantial increase in compliance rates after beginning QI activities, compliance was not well-assessed prior to its implementation. It is possible that a pre-existing “culture of compliance” contributed to the observed success. Effective ways to create and maintain this “culture of compliance” is an important future area of QI research and may vary by country, culture, and institution, and thus may not be as easily achieved in all practice settings.

## Conclusion

Following the implementation of an integrated, multidisciplinary QI strategy for fall prevention in a 520-bed tertiary, acute-care hospital, we observed a 25% reduction in-patient falls with excellent staff compliance. This success, compared to variable outcomes in similar prior studies with lower compliance rates, strongly suggests that staff compliance with QI intervention plays a critical role in its efficacy. High staff compliance was facilitated by multidisciplinary staff involvement in QI activity planning and implementation; daily outcomes monitoring and monthly, ward-specific feedback regarding incidence of falls, consequent injuries, standardized risk assessment rate at admission, and rate of implementation of appropriate intervention; and practical education programs provided to all staff on a regular basis. To reduce the incidence of in-hospital falls, a systematized QI approach that closely involves, encourages, and educates healthcare staff at multiple levels is integral to its effectiveness.

## Competing interests

The authors declare that they have no competing interests.

## Authors' contributions

Study design: SO, MT, AO, OT, GD, TF, MT, RI. Data collection: MT, AO, nursing staff at St. Luke’s International Hospital. Statistical analysis: SO, OT, GD. First draft: SO, Critical revision of the manuscript: SO, OT, GD, MT, AO, TF, MT, RI. Supervision and project guidance: TF. All authors read and approved the final manuscript.

## Funding

This work was supported by the St. Luke’s Life Science Institute Research Fund [grant number: 2008-0-7].

Clinical Research Grant from St. Luke’s Life Science Institute.

## Pre-publication history

The pre-publication history for this paper can be accessed here:

http://www.biomedcentral.com/1472-6963/12/197/prepub
